# Investigation of relationship between vitamin D status and reproductive fitness in Scottish hill sheep

**DOI:** 10.1038/s41598-018-37843-6

**Published:** 2019-02-04

**Authors:** Ping Zhou, Thomas G. McEvoy, Andrew C. Gill, Nicola R. Lambe, Claire R. Morgan-Davies, Emma Hurst, Neil D. Sargison, Richard J. Mellanby

**Affiliations:** 1 Royal (Dick) School of Veterinary Studies and The Roslin Institute, The University of Edinburgh, Easter Bush Veterinary Centre, Roslin, Midlothian, EH25 9RG UK; 2 SRUC (Scotland’s Rural College), King’s Buildings, West Mains Road, Edinburgh, EH9 3JG UK; 3 School of Chemistry, Joseph Banks Laboratories, University of Lincoln, Green Lane, Lincoln, LN6 7DL UK

## Abstract

There is a growing interest in the influence of vitamin D on ovine non-skeletal health. The aim of this study was to explore the relationship between pre-mating vitamin D status, as assessed by serum concentrations of 25-Hydroxyvitamin D [25(OH)D; comprising D_2_ and D_3_] and subsequent reproductive performance of genetically unimproved Scottish Blackface (UBF), genetically improved Scottish Blackface (IBF) and Lleyn ewes kept under Scottish hill conditions. 25-Hydroxyvitamin D_2_ (25(OH)D_2_) and 25-Hydroxyvitamin D_3_ (25(OH)D_3_) concentrations were determined in serum samples harvested in November from ewes grazed outdoors. There were no significant differences in 25(OH)D_2_concentrations amongst the 3 genotypes. Lleyn ewes had significantly higher 25(OH)D_3_ and 25(OH)D concentrations than both Scottish Blackface ewe genotypes, whereas these vitamin D parameters did not differ significantly between the UBF and IBF ewes. Concentrations of 25(OH)D_3_ and 25(OH)D were positively associated with subsequent birth weights of singleton and of twin lamb litters. No significant associations between vitamin D status and number of lambs born or weaned per ewe were found. This study demonstrates that concentrations of cutaneously-derived 25(OH)D_3,_ but not of orally consumed 25(OH)D_2_, differed between breeds. The positive association between ewe vitamin D status and offspring birth weight highlights the need for further investigations.

## Introduction

The two forms of vitamin D, namely D_2_ and D_3_, can be obtained from diet or during sunlight exposure. Vitamin D_2_and its precursor can be found in fungal resources, such as wild mushroom (e.g. *Chantarellus tubaeformis*)^1^, whereas the vitamin D_3_ content of certain animal species, such as wild salmon, is high^2^. During exposure to ultraviolet B radiation (wavelength, 290–315 nm), 7-Dehydrocholesterol in the skin is converted to previtamin D_3_ and then vitamin D_3_. After vitamin D enters the systemic circulation, it is hydroxylated in the liver to the main circulating form, 25-Hydroxyvitamin D (25(OH)D). This metabolite is further hydroxylated in the kidneys to the biologically active form, 1,25-Dihydroxyvitamin D (1α,25-(OH)_2_D)^3^. Compared to 1α,25-(OH)_2_D, the concentration of 25(OH)D is a more reliable indicator for determining vitamin D status, due to its longer half-life, a higher serum concentration and the fact that it is less tightly regulated by parathyroid hormone^4,5^.

Endogenous photobiosynthesis of vitamin D depends on exposure to sunlight which contains sufficient ultraviolet B radiation^6^. The quality and the quantity of ultraviolet B radiation are affected by latitude and season, as when the sun is low in the sky, more ultraviolet B radiation is scattered and absorbed when it travels through the ozone layer, compared to when the sun is directly overhead^7^. In regions where the latitudes are above 39°N, such as the UK (from 49 to 60°N), the low level of ultraviolet B radiation results in no previtamin D_3_ being synthesized from 7-Dehydrocholesterol in human skin during exposure to sunlight, from October to March^6–8^. This could also lead to low vitamin D status in sheep farmed in such high latitude locations, although literature quantifying optimal ultraviolet radiation levels for sheep is difficult to find.

The principal function of vitamin D is the maintenance of skeletal health through regulating the processes of intestinal absorption and renal excretion of calcium and phosphorus, bone formation and mineral mobilization^3^. However, in the last 2 decades, numerous studies have reported that vitamin D deficiency is associated with many non-skeletal health problems, such as autoimmune diseases, hypertension and cancer^9,10^. The presence of vitamin Dreceptors in the reproductive tract of women^11^ and females of other species, such as sheep^12^, goat^13^, mouse^14,15^ and rat^16^, and in ovine male reproductive tracts^17,18^ indicates that vitamin D may influence reproductive performance. Several human studies have found that vitamin D deficiency before and during pregnancy is associated with reduced reproductive success and increased risk of the newborn being small for gestational age, lighter in weight at birth, or having reduced head circumference^19–22^. A recent ovine study suggests a role for vitamin D_3_ in spermatogenesis^18^.

**Figure 1:**
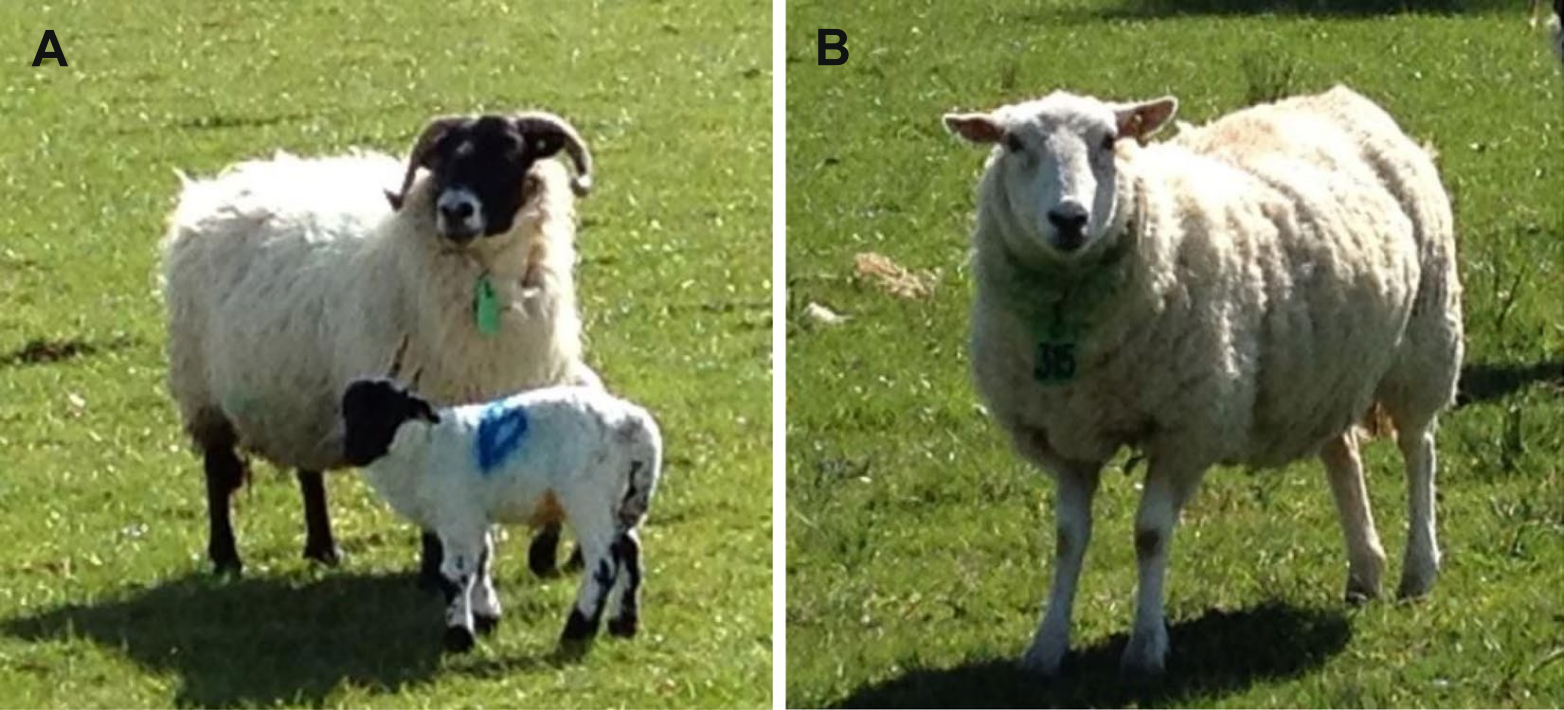
Photographs of Scottish Blackface (**A**) and Lleyn (**B**) ewes at SRUC Hill and Mountain Research Centre (photographs taken by Ping Zhou).

The Scottish Blackface sheep (Fig. 1A) is a hardy hill breed with a white fleece, and mostly black skin and hair on the face and legs, whereas the Lleyn sheep (Fig. 1B) is a lowland/upland prolific breed with white fleece, non-pigmented skin and white hair on its face and legs. The Scottish Blackface is native to Scotland and is the most common breed found in the extensive hill farms of the west coast, where the annual number of sunshine hours is low, during autumn and winter, the monthly average daylight hours ranging from 10 hours 20 minutes in October to 7 hours in December^23^, whereas Lleyn sheep are native to Wales. We investigated the hypothesis that there is a breed-dependent effect on vitamin D status of sheep in North West Scotland (56°N) and that there is a positive relationship between ewe reproductive performance and vitamin D status. To investigate this hypothesis, serum 25(OH)D_2_ and 25(OH)D_3_ concentration of three populations of ewes were determined using high performance liquid chromatography tandem mass spectrometry (HPLC-MS/MS). The relationship between vitamin D status of contrasting genotypes, all in the same flock, and breeding outcomes was investigated.

## Materials and Methods

### Study flock

The experiment was conducted at Scotland’s Rural College (SRUC) Hill and Mountain Research Centre, Crianlarich, Scotland (56°N, 4°W; elevation: 170 to 1025 m; mean annual rainfall: 3,000 mm)^24^. The study flock comprised approximately 200 genetically unimproved Scottish Blackface (UBF) ewes, 200 genetically improved Scottish Blackface (IBF) ewes and 200 Lleyn ewes. The UBF ewes had been selected in each generation to remain close to the average genetic merit in the flock before selection commenced in 1998, while IBF ewes were a lineage progressively selected for superior genetic merit in both ewe and lamb traits^25^. The Lleyn ewes had been selected for high genetic merit since 2010 using the Carcass+ Index (which aims to identify sheep with superior breeding potential for maternal ability, lamb growth and carcase quality) as part of the Signet Sheepbreeder performance recording service (www.signetfbc.co.uk/sheepbreeder/).

Prior to blood sampling (in autumn 2015), the research flock was drawn equally from two ‘pre-study’ management system groups, one group having been managed conventionally (CON), and the other subjected to a Precision Livestock Farming (PLF) management protocol. Each system had different criteria for winter feeding, worming and culling^24,26^. Ewes in each system had shared the same pastures. From mating 2015, the flock was subject to two management systems, both of which used the PLF management approach previously developed^24,26^. Ewes were assigned to either a predominantly ‘Hill Grazing’ or ‘Park Grazing’ management system (Supplementary Fig. S1), with each system having different strategies for using grazing resources and feed supplements.

Ewes were mated in single-sire mating groups. There were 4 single-sire mating groups per genetic line. The mating group size in 2015 ranged from 44 to 50 ewes in each single-sire group. From late-November, ewes were joined with rams selected from their own genotype category for two reproductive cycles of 17 days. Ewes were ultrasound pregnancy-scanned to determine pregnancy status and foetal numbers in mid-February. Two supplementary feeding levels (Supplementary Table S1), either “standard” or more generous “corrective”, were provided in two phases, to help meet ewe intake requirements^27^. The first feeding phase was from early January (6^th^/7^th^ Jan 2016) to scanning (22^nd^/23^rd^ Feb 2016), while the second feeding phase was from scanning to lambing (Supplementary Fig. S1), which began on 15^th^ April. All sheep, depending on weight, body condition score (CS) and pregnancy diagnosis, were assigned to one or other in each phase.

Data were collected for all ewes in the flock and included production (e.g. ewe weight and CS), and reproduction (e.g. pregnancy and lambing) records, as well as individual and group health treatments. Ewes were gathered at pre-mating, mid-pregnancy scanning and pre-lambing. At these handling events, ewes were weighed, and condition scored using a 5 point scoring system^28^. Lambs were tagged within 24 hours after birth, and data were collected on birth weight, sex and litter size. Lamb weights were also recorded at around 8 weeks of age (marking, end of June) and at weaning (mid-August).

All experiments had local ethical approval and were conducted in accordance with UK legislation. The experimental protocols involving animals were approved by the SRUC Animal Welfare and Ethical Review Body.

### Sample collection

In mid-November 2015, during pre-mating handling, blood samples were taken via jugular venepuncture into 6 ml silicone coated red-top blood collection tubes (BD, Plymouth, UK). Ewes were between 1.5 and 6.5 years-old at the time of sampling (Supplementary Table S2). Samples were kept on ice during transportation from farm to the laboratory in Edinburgh for processing. The tubes were centrifuged at 3500 rpm at 4 °C for 10 minutes. Serum was then removed into 2 ml screw capped micro-tubes. The 0.5 ml serum aliquots were stored at −20 °C overnight, then stored at −80 °C until analysis.

### Determination of 25(OH)D_2_ and 25(OH)D_3_ concentrations

Blood samples of 88 ewes per genotype were analysed to determine pre-mating ewes’ 25(OH)D_2_ and 25(OH)D_3_ serum concentrations.

#### Calibration standards

Eight calibration standards were freshly prepared, by adding 20 µl of 25(OH)D_2_ stock solution (5 µg/ml in ethanol; Sigma-Aldrich, UK) and 30 µl of 25(OH)D_3_ stock solution (5 µg/ml in ethanol; Sigma-Aldrich, UK) into 1 ml artificial serum [50 mg bovine serum albumin (Sigma-Aldrich, UK) were dissolved in 1 ml of phosphate buffered saline (prepared in house)], then 1 in 2 serial dilution with artificial serum. The concentrations of calibration standards were 230.8, 115.4, 57.7, 28.9, 14.4, 7.2, 3.6 and 1.8 nmol/l for 25(OH)D_2_; and 356.6, 178.3, 89.2, 44.6, 22.3, 11.2, 5.6 and 2.8 nmol/l for 25(OH)D_3_. These calibration standards were used to generate standard curves for quantification of the concentration of 25(OH)D_2_ and 25(OH)D_3_ in sheep serum by HPLC-MS/MS analysis.

#### Sample preparation

After serum samples (0.5 ml) were thawed at room temperature, 100 µl of each sample, or calibration standard, was spiked in a 1.5 ml microtube with 2 µl of 6,19,19-d_3_-25(OH)D_2_ (1.78 µmol/l; Sigma-Aldrich, UK) and 2 µl of 23,24,25,26,27-^13^C_5_-25(OH)D_3_ (2.47 µmol/l; Sigma-Aldrich, UK), as internal standards. After adding 20 µl of 1 M NaOH, each serum sample or calibration standard was then protein precipitated by the addition of 200 µl of acetonitrile^29^. The supernatant of the serum sample or the calibration standard was purified by solid phase extraction using a Discovery DSC-18 SPE-96 Plate (bed weight: 25 mg/well; Sigma-Aldrich, UK). Briefly, the plate was activated with 3 ml of ethyl acetate, 3 ml of methanol and 3 ml of distilled water. After addition of a mixture of supernatant (approximately 300 µl) from protein precipitation and 1 ml 0.4 M K_2_HPO_4_, the plate was washed with 3 ml of distilled water and 2 ml of 40% methanol sequentially and eluted with 1.5 ml of acetonitrile^29^. After evaporating to dryness, samples were derivatized by 2 additions of 25 µl of 0.1 mg/ml DMEQ-TAD (4-[2-(3,4-Dihydro-6,7-dimethoxy-4-methyl-3-oxo-2-quinoxalinyl)ethyl]−3*H*-1,2,4-triazole-3,5(4 *H*)-dione; Abcam, UK) in ethyl acetate^30^. After evaporation to dryness, derivatized extracts were reconstituted in 25 µl of 60:40 (vol:vol) methanol and 0.1% formic acid:water for HPLC-MS/MS analysis.

#### HPLC-MS/MS analysis

The HPLC-MS/MS analyses were conducted using an UltiMate 3000 HPLC system interfaced to an amaZon ETD tandem mass spectrometer (Bruker Daltonics, Bremen, Germany). Chromatographic separations were achieved using an ACE UltraCore 2.5 SuperC18 column (75 × 2.1 mm, 2.5 µm; Advanced Chromatography Technologies, UK), maintained at 40 °C. Gradient elution was performed (Supplementary Table S3), with the mobile phase consisting of 10 mM ammonium formate (Fisher Scientific) with 0.15% formic acid (buffer A) and methanol with 0.1% formic acid (buffer B). The elution was detected using multiple reaction monitoring with positive electrospray ionisation (Supplementary Table S4 and Supplementary Fig. S2). The total runtime was 12 min per sample.

HPLC-MS/MS analyses of sheep serum samples were conducted on different dates on batches of samples comprised of 8 calibration standards and 24 sheep serum samples. Quantitation was carried out using QuantAnalysis 2.0 software (Bruker Daltonics, Bremen, Germany). The standard curve was generated based on the ratio of the peak area of the standard to that of the corresponding internal standard (Supplementary Table S5). Method performance, i.e. injection carryover, sample preparation recovery, intra-assay coefficient of variation and inter-assay coefficient of variation were determined (Supplementary Table S6).

### Statistical analysis

Statistical analyses were conducted using GenStat 16 statistical package (VSN International Ltd. UK). Generalized Linear Models (GLM) were used to investigate the effects of multiple independent variables. Stepwise regression was used to determine the fixed effects and relevant covariates to include in the final model for each response variate. These models were then applied in Linear Mixed Models (LMM), alongside appropriate random effects, to investigate: i) differences in vitamin D concentrations between breeds and among genotypes; and ii) any associations between vitamin D status and ewe breeding outcomes, as well as ewe litter weight at birth, marking and weaning (see Supplementary Table S7 for summary). Statistical significance was defined as P < 0.05. When model terms were significant, pairwise Student’s t-tests were performed to test for significant differences between different levels of each factor.

To investigate differences in vitamin D concentrations [25(OH)D_2_, 25(OH)D_3_ and total 25(OH)D (addition of 25(OH)D_2_ and 25(OH)D_3_)] between breeds and amongst genotypes, the ewe breed/genotype, ewe age, pre-study system, ewe pre-mating weight, ewe pre-mating CS, sire of the ewe, number of lambs weaned and weaned litter weight in the last breeding cycle were the factors considered in the maximal GLM models.

In the GLM models used to examine ewe breeding outcomes, the number of lambs born in 2016 and the number of lambs weaned that year, were tested, in turn, as response variates, whilst 25(OH)D_2_/25(OH)D_3_/25(OH)D concentration, ewe genotype, ewe age, management system (Hill grazing or Park grazing), pre-study system (CON or PLF), ewe pre-mating weight, ewe pre-mating CS, number of lambs weaned in the last breeding cycle (i.e. in 2015) and first winter feeding level were fitted in the maximal models.

When analysing the association of vitamin D status with litter weight at birth, marking and weaning, 25(OH)D_2_/25(OH)D_3_/25(OH)D concentration, ewe genotype, ewe age, management system, pre-study system, first winter feeding level, ewe pre-mating weight, ewe pre-mating CS and ram group were the factors considered in the maximal models in the GLM analyses. These analyses were conducted separately for single- and twin-bearing ewes.

The final fixed models, selected by stepwise regression, and random effects, fitted for each response variate, are shown in Supplementary Table S7. The random effects were either ‘batch’ for testing vitamin D status between breeds and amongst genotypes, or ‘batch and ram group’ for the rest of the LMMs. Batch represented HPLC-MS/MS analysis date. Ram group identified the relevant single sire mating group.

## Results

### Pre-mating (November 2015) vitamin D concentrations

Analysis of two hundred and sixty-four sheep serum samples for pre-mating ewes’ 25(OH)D_2_ and 25(OH)D_3_ concentrations indicated that the latter differed between breeds. The unadjusted average serum concentrations of 25(OH)D_2_, 25(OH)D_3_ and total 25(OH)D for the UBF, IBF and Lleyn ewes are shown in Table 1.

**Table 1:** The unadjusted concentrations (mean ± standard error of mean) of 25(OH)D_2_, 25(OH)D_3_ and 25(OH)D for the 3 genotypes of ewes.

Genotype	Sample size	25(OH)D_2_ (nmol/l)	25(OH)D_3_ (nmol/l)	25(OH)D (nmol/l)
UBF	83	18.6 ± 0.7	19.4 ± 0.9	38.0 ± 1.3
IBF	88	20.0 ± 0.7	19.6 ± 0.9	39.6 ± 1.4
Lleyn	88	20.0 ± 0.6	24.3 ± 1.1	44.3 ± 1.4
Average	—	19.5 ± 0.4	21.1 ± 0.6	40.7 ± 0.8

The results for five UBF ewes were excluded from the data reported here, as either their measured serum 25(OH)D_3_ concentration, or both 25(OH)D_2_ and 25(OH)D_3_ concentrations were beneath the lower quantification limit (7.2 and 5.6 nmol/l for 25(OH)D_2_ and 25(OH)D_3_, respectively) of the assay as applied in the current experiment.

**Figure 2:**
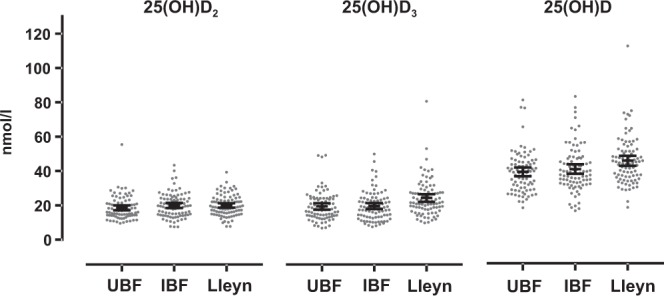
The pre-mating concentration of 25(OH)D_2_, 25(OH)D_3_ and 25(OH)D of individual ewe samples for the 3 genotypes. Short black lines show the mean with 95% confidence interval.

Eighteen ewes (6.9%) had 25(OH)D concentrations <25 nmol/l, 238 ewes (91.9%) had concentrations between 25 and 75 nmol/l, and 3 ewes (1.2%) had concentrations >75 nmol/l (Fig. 2).

**Figure 3:**
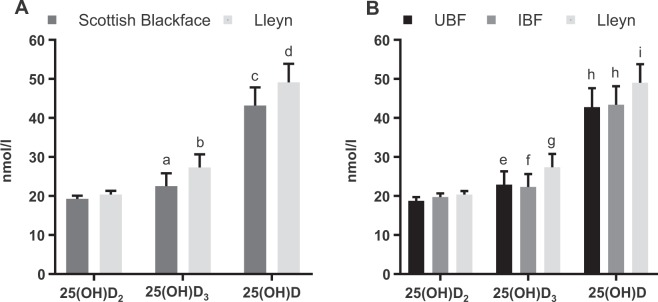
The predicted means ± standard error of 25(OH)D_2_, 25(OH)D_3_ and total 25(OH)D concentrations (**A**) between breeds and (**B**) among the 3 genotypes. Significant differences denoted as follows: ^ab^P < 0.001; ^cd^P < 0.001; ^eg^P < 0.01; ^fg^P < 0.001; ^hi^P < 0.01.

Serum 25(OH)D_2_ concentrations did not differ among either breed (P = 0.188) or genotype (P = 0.263) after adjusting for age and ewe pre-mating weight (Fig. 3). Serum concentrations of 25(OH)D_2_ also were not significantly associated with ewe pre-mating weight or age (P > 0.05).

When breed, ewe pre-mating weight, number of lambs weaned and weaned litter weight in the last breeding cycle were accounted for in the LMM, there was a significant difference in 25(OH)D_3_ concentration between the Scottish Blackface and Lleyn ewes (P < 0.001; Fig. 3A). When genotype (n = 3) was fitted, rather than just breed, the LMM on 25(OH)D_3_ concentration among the genotypes showed that Lleyn ewes had significantly higher levels of 25(OH)D_3_ than UBF and IBF ewes (P < 0.01 and P < 0.001, respectively; Fig. 3B), whereas the concentration of this vitamin D metabolite did not differ significantly between UBF and IBF ewes (P > 0.05; Fig. 3B). Ewe pre-mating weight was positively associated with 25(OH)D_3_ concentration (P = 0.014 and P = 0.013, respectively, when either breed or genotype was fitted in the model).

Similarly, Lleyn ewes had a significantly higher total 25(OH)D concentration than UBF and IBF ewes (P < 0.01 and P < 0.01, respectively; Fig. 3B), whereas there was no significant difference in total 25(OH)D concentration between UBF and IBF ewes (P > 0.05). Ewe pre-mating weights were positively associated with 25(OH)D concentrations in the models with breed (P = 0.015) and genotype (P = 0.017) fitted, respectively.

### Pre-mating vitamin D status and subsequent breeding outcome

**Figure 4:**
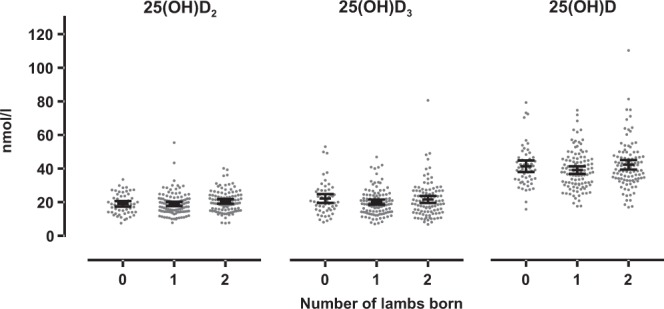
Pre-mating 25(OH)D_2_, 25(OH)D_3_ and 25(OH)D concentrations recorded for ewes (all 3 genotypes) that subsequently (in 2016) gave birth to 0, 1 or 2 lambs. Short black lines show the means and 95% confidence intervals.

The unadjusted pre-mating 25(OH)D_2_, 25(OH)D_3_ and 25(OH)D concentrations in UBF, IBF and Lleyn ewes grouped according to subsequent litter sizes at birth and at weaning, are presented in Table 2 and Fig. 4. When ewe genotype, age, pre-study system, management system, ewe pre-mating weight, the number of lambs weaned in the last breeding cycle and first winter feeding level were included in the three LMM models, there were no significant differences in serum 25(OH)D_2_ (P = 0.06), 25(OH)D_3_ (P = 0.57) and 25(OH)D (P = 0.534) concentrations amongst ewes that, in 2016, were barren or had singles or had twins.

**Table 2:** The unadjusted pre-mating serum concentrations (nmol/l) of 25(OH)D_2_, 25(OH)D_3_ and 25(OH)D [mean ± standard error of the mean (sample size)] in ewes categorised on the basis of number of lambs born and number of lambs weaned in 2016 for the 3 genotypes.

		Number of lambs born			Number of lambs weaned		
		0	1	2	0	1	2
25(OH)D_2_ (nmol/l)	UBF	18.4 ± 1.5 (17)	18.2 ± 1.3 (39)	19.3 ± 1.1 (27)	18.9 ± 1.3 (20)	18.4 ± 1.2 (43)	18.8 ± 1.2 (20)
	IBF	18.9 ± 1.4 (18)	19.1 ± 1.2 (34)	21.4 ± 1.2 (36)	18.6 ± 1.3 (24)	19.3 ± 1.1 (36)	22.0 ± 1.4 (28)
	Lleyn	19.7 ± 1.4 (19)	19.6 ± 1.0 (33)	20.5 ± 1.1 (36)	19.8 ± 1.2 (24)	19.7 ± 1.1 (31)	20.4 ± 1.2 (33)
25(OH)D_3_ (nmol/l)	UBF	22.5 ± 2.4 (17)	17.8 ± 0.9 (39)	19.7 ± 2.0 (27)	22.7 ± 2.0 (20)	17.6 ± 0.8 (43)	19.8 ± 2.6 (20)
	IBF	19.4 ± 2.3 (18)	18.3 ± 1.0 (34)	20.9 ± 1.6 (36)	19.7 ± 1.7 (24)	18.6 ± 1.1 (36)	20.8 ± 1.9 (28)
	Lleyn	24.6 ± 2.2 (19)	24.7 ± 1.8 (33)	23.9 ± 1.9 (36)	24.7 ± 2.0 (24)	24.4 ± 1.8 (31)	24.0 ± 2.1 (33)
25(OH)D (nmol/l)	UBF	41.0 ± 3.0 (17)	36.0 ± 1.7 (39)	38.9 ± 2.5 (27)	41.6 ± 2.6 (20)	36.0 ± 1.6 (43)	38.6 ± 3.2 (20)
	IBF	38.3 ± 3.0 (18)	37.3 ± 1.7 (34)	42.4 ± 2.5 (36)	38.4 ± 2.4 (24)	37.9 ± 1.7 (36)	42.8 ± 3.0 (28)
	Lleyn	44.3 ± 2.9 (19)	44.3 ± 2.2 (33)	44.3 ± 2.5 (36)	44.6 ± 2.4 (24)	44.1 ± 2.4 (31)	44.4 ± 2.7 (33)

Pre-mating 25(OH)D_2_ (P = 0.130) concentration was not associated with number of lambs weaned, when this trait was adjusted for genotype, age, management system, pre-study system, ewe pre-mating weight, ewe pre-mating CS, number of lambs weaned in the last breeding cycle and first winter feeding level. In addition, the LMM models with 25(OH)D_3_ and 25(OH)D concentrations, fitted with genotype, management system, pre-study system, ewe pre-mating weight, ewe pre-mating CS, number of lambs weaned in the last breeding cycle and first winter feeding level, showed that 25(OH)D_3_ (P = 0.287) and 25(OH)D (P = 0.978) status at pre-mating was not a significant determinant of the number of lambs weaned in August 2016.

### Pre-mating vitamin D status and subsequent ewe litter weights at birth, marking and weaning

Among the 259 ewes in this study, 54 (21%) did not produce any lambs; the remainder produced one or two lambs. The overall lambing percentage was 117%, with 112% for UBF ewes, 120% for IBF ewes and 119% for Lleyn ewes.

#### Ewes that produced singleton lambs

One hundred and six ewes gave birth to singletons. The associations between the pre-mating vitamin D status of ewes and their subsequent singletons’ weights at birth, marking and weaning were investigated, based on all those ewes, including 13 ewes (12%) that lost their lambs between birth and weaning.

**Figure 5:**
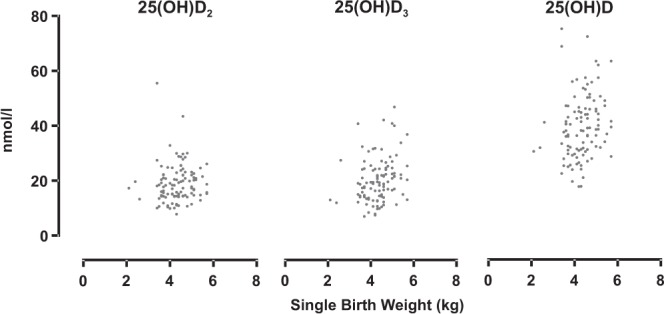
Unadjusted pre-mating 25(OH)D_2_, 25(OH)D_3_ and 25(OH)D concentrations and subsequent lamb birth weights (kg) of single-bearing ewes (3 genotypes combined).

There was no relationship between serum 25(OH)D_2_ concentration and the birth weight of singletons (P = 0.355; Fig. 5), after accounting for the effects of genotype, management system, ewe pre-mating weight and first winter feeding level, whereas 25(OH)D_3_ and total 25(OH)D concentrations were positively associated with lamb birth weight of singletons (P = 0.008 and P = 0.017, respectively; Fig. 5), when genotype, management system and first winter feeding level were accounted for in the LMM models.

For the singleton marking weights, when genotype, management system, first winter feeding level and ewe pre-mating weight were included in the LMM model, none of the 25(OH)D_2_ (P = 0.246), 25(OH)D_3_ (P = 0.408), or 25(OH)D (P = 0.196) concentrations proved to be a significant factor. When genotype, first winter feeding level and ewe pre-mating weight were considered in the LMM models, 25(OH)D_2_ (P = 0.542), 25(OH)D_3_ (P = 0.722) and 25(OH)D (P = 0.535) status had no effect on lamb weaning weight.

#### Ewes that produced twin lambs

Ninety-nine ewes gave birth to twins. The associations between the pre-mating vitamin D status of ewes and their subsequent twin litter weights at birth, marking and weaning were investigated, based on all 99 ewes, including 1 ewe (1%) having no lamb weaned and 17 (17%) ewes having one lamb weaned.

**Figure 6:**
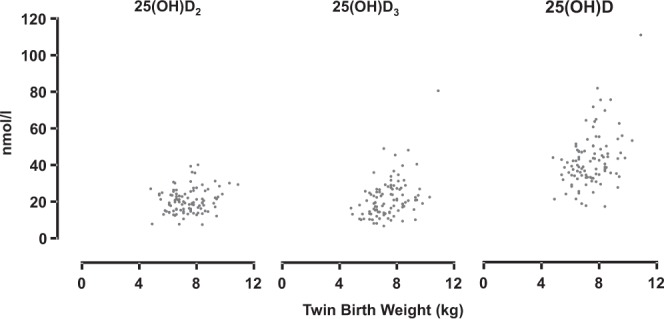
Unadjusted pre-mating 25(OH)D_2_, 25(OH)D_3_ and 25(OH)D concentrations and subsequent litter birth weights (kg) of twin-bearing ewes (3 genotypes combined).

When genotype, age, pre-study system, ewe pre-mating weight and ewe pre-mating CS were considered in the LMM model, the serum 25(OH)D_2_ concentration was not a significant determinant of litter birth weight (P = 0.128; Fig. 6). Ewe pre-mating serum 25(OH)D_3_ and 25(OH)D concentrations were positively associated with litter birth weight of twins (P < 0.001 and P < 0.001, respectively; Fig. 6), when genotype, management system, ewe pre-mating weight and ewe pre-mating CS were fitted in the LMM models.

At marking, when genotype, management system, first winter feeding level, ewe pre-mating weight and ewe pre-mating CS were included in the LMM, none of 25(OH)D_2_ (P = 0.540), 25(OH)D_3_ (P = 0.265), and 25(OH)D (P = 0.267) ewe pre-mating serum concentrations showed a significant association with the litter marking weight of the ewes’ twin offspring. When genotype, management system, first winter feeding level, ewe pre-mating weight and ewe pre-mating CS were adjusted for in the LMM analyses, none of 25(OH)D_2_ (P = 0.418), 25(OH)D_3_ (P = 0.355), and 25(OH)D (P = 0.297) had a significant association with the ewes’ twin litter weaning weights.

## Discussion

This study has identified a clear relationship between genotype and vitamin D_3_, but not D_2_, status in a commercially operated sheep farm. Sheep obtain vitamin D_2_ from their diet^8^. In this study, the sheep shared the same pastures, and thus the fact that serum 25(OH)D_2_ concentration did not differ significantly amongst the 3 genotypes indicates that genotype was not a determinant of vitamin D_2_ status within the flock. The concentrations of 25(OH)D_2_ recorded for the UBF, IBF and Lleyn ewes (means: 18.6 to 20.0 nmol/l; Table 1) were similar to those of adult ewes (1–6 years old) reported by Handel *et al*.^31^, but lower than results reported by Kohler *et al*.^32^ from Switzerland (means: 36.7 and 26.0 nmol/l for East Friesian lactating milk sheep kept at altitude of 2,000–2,600 and 400 m for 12 weeks during summer, respectively). Differences could be due to the different blood sampling seasons and contrasting geographical regions with different vegetation in the pasture, which influence herbage vitamin D_2_ content^33^.

The sheep serum concentrations of 25(OH)D_3_ in this study indicate the extent to which vitamin D has been obtained via endogenous photobiosynthesis, since they did not receive any supplementary foodstuffs which contained vitamin D_3_, for at least 5 months prior to blood sampling. Lleyn ewes had significantly higher 25(OH)D_3_ concentrations, therefore the black heads and legs of the Blackface ewes might have compromised vitamin D_3_ photobiosynthesis in those black hair covered areas that are contiguous with dark skin pigmentation^34^. The efficiency of the conversion of 7-Dehydrocholesterol to previtamin D_3_ in Scottish Blackface ewes might be compromised because melanin in the pigmented skin competes with 7-Dehydrocholesterol for ultraviolet B photons^35^, although the face and limbs might not be the main sites for vitamin D photobiosynthesis in sheep^8^. All ewes were shorn in late June only, so had full fleece cover prior to blood sampling. Breed differences in terms of 25(OH)D_3_ could also mean that the Lleyn ewes have a superior capability to retain vitamin D_3_ as biologically inert forms such as tachysterol and lumisterol in their body than their BF counterparts^35^.

Reports of rickets outbreaks in sheep in Scotland^36,37^ indicate that vitamin D deficiency has occurred in geographical locations close to our study site. In regions above latitudes of 55°N, sunlight only provides sufficient intensity of ultraviolet radiation (290–315 nm) to induce a conversion of 7-Dehydrocholesterol to previtamin D_3_^38^ between mid-March and mid-September^39^. In the Handel *et al*. (2016) study^31^, the serum 25(OH)D_3_ concentrations in the Soay sheep from St Kilda (57.8°N 8.6°W) were measured in samples taken in August, whereas in this study (56°N 4°W) sheep samples were taken in mid-November. This might explain why the serum 25(OH)D_3_ concentrations of sheep in this study were much lower than those of Soay sheep (unadjusted concentration: 21.1 *vs*. 42.8 nmol/l, respectively)^31,39^.

Although the vitamin D content in grass is limited, and it is argued that grazing animals obtain vitamin D mainly from endogenous photobiosynthesis^34^, our results show that 25(OH)D_2_ contributed nearly half of the total 25(OH)D concentration for all three genotypes at the November sampling timepoint. Nevertheless, Lleyn ewes showed significantly higher total 25(OH)D concentrations than Scottish Blackface ewes. This observation of a breed effect was in agreement with Willems *et al*.^40^, albeit that different sheep breeds were investigated. The concentration of 25(OH)D has also been reported to be positively associated with skin thickness, which decreases with age^41^. Unlike Handel *et al*.^31^, the current study did not find an age-related effect on the vitamin D metabolites investigated, probably due to a narrower age range of ewes (from 1.5 to 6.5 years old at blood sampling time) investigated, whilst Handel *et al*.^31^ studied a more age-diverse (from 0.5 to 13 years old) population of feral sheep. The age range considered in this study more or less corresponds with and affirms the ‘Adult’ category (combining ages from 1 to 6 years) used by Handel *et al*.^31^ to distinguish that cohort of Soay ewes from same-breed lambs and geriatric sheep in their study.

In the current study, the breeding outcomes (i.e. number of lambs born or weaned) were not associated with pre-mating ewe status in respect of the vitamin D metabolites investigated. Nevertheless, 9 ewes that lost foetus(es) between scanning and lambing (based on scanning results), had lower concentrations for one or both vitamin D metabolites investigated, compared to the corresponding average unadjusted vitamin D concentrations (Table 2). This indicates that vitamin D might impact on foetal survival in the uterus. Of course, other factors, such as breed differences^42^, ewe age^43^ or poor nutrition^44^, could also contribute and larger studies would be required to fully assess the effect of vitamin D status on foetal survival. In contrast to our findings, the Soay sheep study demonstrated that vitamin D status (August, not November as in our case) was positively associated with annual reproductive success in ewes, i.e. the number of their lambs that survived to one year old^31^. According to the benchmark (vitamin D deficiency: <25 nmol/l, insufficient: 25–75 nmol/l, sufficient: 75–150 nmol/l) recommended for most species^8^, the majority of the ewes in our study were vitamin D insufficient, and in general, their 25(OH)D levels were lower than those reported for Soay sheep in St Kilda, Scotland^31^. However, our sheep were sampled in November, not August, and the general reproductive competence suggests they were not severely compromised^45^. We suggest that these ewes have adapted well to the environmental conditions in north west Scotland, and that their adaptation could include capability to thrive with a vitamin D status below the ‘sufficiency’ range posited by Nemeth *et al*.^8^, at least for part of the year^46,47^, as a prerequisite for survival.

Ewes in our study that received the standard feeding level in the first winter feeding period had better body condition at that stage, compared to those with poorer body condition that received the corrective feeding level. These ewes with better condition scores in winter then went on to have greater litter sizes at birth and weaning in the subsequent spring and summer, respectively than their counterparts with relatively poorer body conditions. This is plausible and not unexpected as ewes with good body condition would have increased ovulation rates, reduced risk of embryo loss^48,49^, and would produce lambs with heavier birth weights^50,51^.

In this study, ewes with higher serum 25(OH)D_3_ and total 25(OH)D concentrations produced heavier lambs for both singleton and twin litter categories. These findings were in line with previous reports of vitamin D status effects in pregnant women^19,22,52,53^. One possible explanation is that high 25(OH)D status could promote local metabolism from 25(OH)D to the active form of vitamin D, 1α,25(OH)_2_D, in the placenta, which supports placental development in early pregnancy^54^, and in turn enhances placental transportation of calcium for foetal growth^55,56^. However, this correlation (improved vitamin D status associated with lower incidence of low infant birthweight) has been a controversial issue in human studies, as several authors report different results^57–59^. Environmental conditions are more difficult to standardise in human studies, or those involving wild or feral animals, compared to those conducted on livestock within a research farm environment, which could account for differences in findings across trials and species. Our results suggest that ewes having higher pre-mating vitamin D status might, if there is a pay-off in terms of foetal growth, be favoured in a hill environment, where lamb birth weight is an important determinant of lamb survival in harsh conditions^60,61^. Equally, our results suggest that aforementioned ‘sufficient’ concentrations^8^ may be in excess of what high latitude- and altitude-adapted sheep either could accrue or in fact need to reproduce competently and successfully.

Our results showed that ewes’ pre-mating vitamin D status was associated with the lamb birth weight, but not the weight during early growth (marking at around 8 weeks old) or at weaning (around 18 weeks of age). In the early postnatal stage, especially between birth and marking, lamb growth relies mainly on the mother’s milk production^62^, which apparently was not influenced by pre-mating vitamin D status of ewes in the current study.

## Conclusion

Our findings show that the 25(OH)D_3_ and total 25(OH)D concentrations in Lleyn ewes were significantly higher than those in both unimproved and improved Scottish Blackface ewes kept at a Scottish commercial hill farm. The vitamin D status in the 3 genotypes did not have any effects on the number of lambs born or weaned per ewe. The concentrations of 25(OH)D_3_ and 25(OH)D were positively associated with litter weight for singleton and twin lambs at birth, but not at marking or weaning. The relationships between vitamin D status and reproductive outcomes in sheep are deserving of further study.

## Supplementary information

Investigation of relationship between vitamin D status and reproductive fitness in Scottish hill sheep

## Data Availability

Data supporting the findings of the current study are available from the corresponding author on reasonable request.
