# Supplementary material for: Investigation of relationship between vitamin D status and reproductive fitness in Scottish hill sheep
Source: Sci Rep. 2019 Feb 4;9:1162. doi: 10.1038/s41598-018-37843-6 (PMC6362214; doi:10.1038/s41598-018-37843-6)
Supplement: Supplementary file 1 — Investigation of relationship between vitamin D status and reproductive fitness in Scottish hill sheep [file 41598_2018_37843_MOESM1_ESM.pdf]

Supplementary File

Investigation of relationship between vitamin D status and reproductive fitness in Scottish hill sheep

Ping Zhou[1,2], Thomas G McEvoy[2], Andrew C Gill[1,3], Nicola R Lambe[2], Claire R Morgan-Davies[2], Emma Hurst[1], Neil D Sargison[1], Richard J Mellanby[1*]

[1]Royal (Dick) School of Veterinary Studies and The Roslin Institute, The University of Edinburgh, Easter Bush Veterinary Centre, Roslin, Midlothian, EH25 9RG UK.
[2]SRUC (Scotland's Rural College), Kings Buildings, West Mains Road, Edinburgh, EH9 3JG UK.
[3]School of Chemistry, Joseph Banks Laboratories, University of Lincoln, Green Lane, Lincoln, LN6 7DL UK.

[*] Corresponding author: Royal (Dick) School of Veterinary Studies and The Roslin Institute, Division of Veterinary Clinical Studies, The University of Edinburgh, Hospital for Small Animals, Easter Bush Veterinary Centre, Roslin, Midlothian, EH25 9RG UK.
Email address: Richard.Mellanby@ed.ac.uk (Richard J Mellanby)
Telephone: +44 (0)131 650 7650

Supplementary Figure S1.

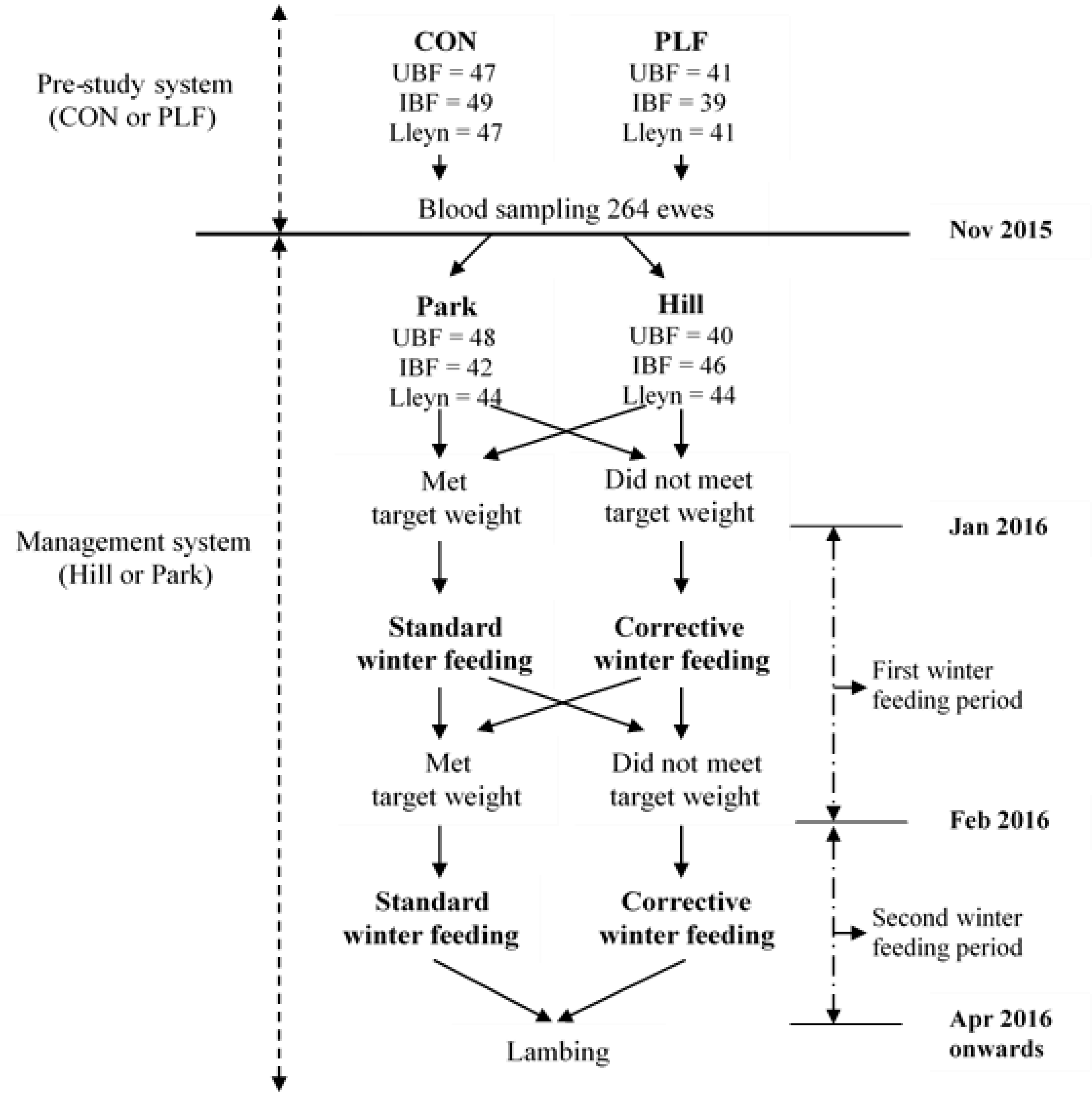

Supplementary Figure S1. Flowchart of management systems and winter supplementary feeding periods in the experiment. The target weight was calculated for every individual ewe.

Supplementary Figure S2.

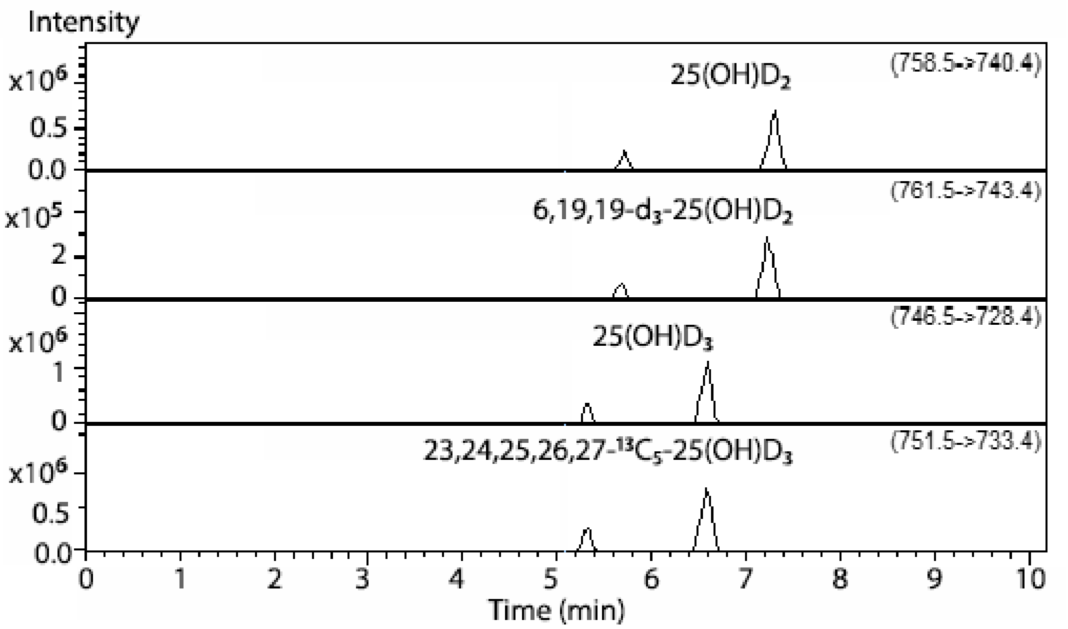

Supplementary Figure S2. The chromatogram of a standard solution of 25(OH)D_2 (28.85 nmol/l) and 25(OH)D_3 (44.58 nmol/l). Multiple reaction monitoring was at 758.5>468.1 for 25(OH)D_2, and at 746.5>468.1 for 25(OH)D_3.

Supplementary Table S1.

**Protein (%)**	18	**Sodium (%)**	2.7
**Urea (%)**	2.5	**Zinc (mg/kg)**	500
**Oil (%)**	7	**Manganese (mg/kg)**	250
**Fibre (%)**	3	**Iodine (mg/kg)**	20
**Ash (%)**	19	**Cobalt (mg/kg)**	2.5
**ME (MJ/kg DM)**	13	**Selenium (mg/kg)**	2.5
**Calcium (%)**	3.3	**Vitamin A (iu/kg)**	25,000
**Phosphorus (%)**	0.27	**Vitamin D_3 (iu/kg)**	5,000
**Magnesium (%)**	2	**Vitamin E (iu/kg)**	500

Supplementary Table S1. Product specification of Rumevite Sheep Super Energy plus Fish Oil. Rumenco Rumevite Sheep Super Energy plus Fish Oil mineral blocks were supplied during winter supplementary feeding periods in 2016.

<u>Supplementary Table S2.</u>

	Ewe age at sampling (years)					
	**1.5**	**2.5**	**3.5**	**4.5**	**5.5+**	**Total**
**UBF**	15	43	11	9	10	88
**IBF**	15	39	15	6	13	88
**Lleyn**	19	33	19	2	5	88

Supplementary Table S2. The distribution of ewes by age at blood sampling for the 3 genotypes.

Supplementary Table S3.

**LC parameters**	
Injection volume	5 µl
Flow rate	200 µl/min
Column temperature	40 °C
Injection needle rinse	20% Methanol
Mobile phase gradient:	
0 min	Sample injection
0 min to 1 min	20% B to 72% B
1 min to 7.5 min	72% B
7.5 min to 9 min	72% B to 100% B
9 to 9.1 min	100% B to 20% B
9.1 to 10 min	20% B
Equilibration	2 min of 20% B
Total run time	12 min
**MS parameters**	
Electrospray ionisation mode	Positive
Capillary current	4500 V
Nebulizer gas	Nitrogen; 16 psi
Dry gas flow rate	8.0 L/min
Dry gas temperature	220°C

Supplementary Table S3: Instrumentation set-up for HPLC-MS/MS analysis. The top panel was for liquid chromatography, while the bottom panel was for mass spectrometry.

Supplementary Table S4.

Analyte	Mass transition	Fragmentation cut-off *m/z* and fragmentation amplitude, *v*	Retention time (min)
**25(OH)D_2**	758.5>740.4>468.1	1st precursor: 163/0.90; 2nd precursor: 167/0.53	5.7 & 7.3
**6,19,19-d_3-25(OH)D_2**	761.5>743.4>471.1	1st precursor: 184/0.95; 2nd precursor: 167/0.53	5.7 & 7.3
**25(OH)D_3**	746.5>728.4>468.1	1st precursor: 185/0.70; 2nd precursor: 164/0.80	5.4 & 6.6
**23,24,25,26,27-$^{13}C_5$-25(OH)D_3**	751.5>733.4>468.1	1st precursor: 185/0.70; 2nd precursor: 164/0.80	5.4 & 6.6

Supplementary Table S4: The mass transitions and the optimised conditions for the selected MS/MS method for detecting 25(OH)D_2 and 25(OH)D_3 as well as their corresponding internal standards (i.e. 6,19,19-d_3-25(OH)D_2 and 23,24,25,26,27-$^{13}C_5$-25(OH)D_3, respectively).

The major characteristic ions for 25(OH)D_2 and 25(OH)D_3 derivatives were at a mass/charge (m/z) ratio of 758.5 and 746.5, respectively. The optimised conditions (fragmentation cut-off and amplitude) for these 2 vitamin D metabolites were detected using a direct infusion method with a syringe pump. Under optimised HPLC-MS/MS conditions, each of 25(OH)D_2 and 25(OH)D_3 derivatives generated 2 main fragments, which were a DMEQ-TAD fragment with m/z ratio of 247.0 and A-ring/DMEQ-TAD with m/z ratio of 468.1. Both fragment ions were used in the multiple reaction monitoring during tandem mass spectrometry. Two isomers, 6R and 6S were produced during derivatisation, thus there were 2 peaks for each analyte in the resultant chromatogram.

Supplementary Table S5.

Analyte	r^2	LLOD (nmol/l)	LLOQ (nmol/l)	Calibration range (nmol/l)
**25(OH)D_2**	0.998	3.6	7.2	1.8 – 230.8
**25(OH)D_3**	0.996	5.6	5.6	2.8 – 356.6

Supplementary Table S5: Details of calibration curves.

The derivative of 25(OH)D with DMEQ-TAD reagent comprised 6S and 6R isomers. The major product, 6S isomer generated a larger peak, which was used for quantifying the quantity of the analyte. The calibration curve was constructed by using the ratio of the larger peak area of the standard to that of the corresponding internal standard (i.e. 25(OH)D_2-d_3 or $^{13}C_5$-25(OH)D_3). The correlation coefficients (r^2) were calculated from the means of 8 calibration curves. Lower limit of detection (LLOD) and lower limit of quantification (LLOQ) were determined by identifying the lowest standard contained within the standard curve that had a minimum signal to noise ratios of 5:1 and 10:1, respectively, and for which observed concentration was within 30% of intent.

Supplementary Table S6.

Analyte	Injection carryover (%)	Recovery rate (%)	Intra assay coefficient variation (%)	Inter assay coefficient variation (%)
**25(OH)D_2**	0.01	63	7.5	17.3
**25(OH)D_3**	0.02	54	6.9	15.1

Supplementary Table S6: Method validation of the HPLC-MS/MS analysis of 25(OH)D_2 and 25(OH)D_3 in sheep serum samples.

Injection carryover was determined by comparing the peak areas of the analytes in the validation run of the highest concentration standard with the corresponding areas in the bland [60:40 (vol:vol) methanol with 0.1% formic acid:water], that was analysed immediately afterwards. The injection carryovers reported here were the means of 8 runs. The recovery rate of sample preparation was examined by comparison of the artificial serum spiked with stock standards (and then put through the sample preparation procedures demonstrated in the method) with the post-extracted samples. The post-extracted samples were generated by spiking the same amount of stock standards into a tube that contained dried artificial serum which had already gone protein precipitation and solid phase extraction. Three concentrations [233.0, 116.5 and 58.3 nmol/l for 25(OH)D_2; 234.0, 120.0 and 60.0 nmol/l for 25(OH)D_3] of samples were prepared in duplicates with internal standards. The total peak areas of 6S and 6R isomers were used for calculating the recovery rate. Each sample or calibration standard was analysed in duplicate throughout HPLC-MS/MS analysis. Intra-assay coefficient variation was obtained from 5 runs (consisting of the results of 88 sheep serum samples). Inter-assay coefficient variation was determined on the basis of results from four Scottish Blackface sheep serum samples analysed in 3 consecutive runs.

Supplementary Table S7. Summary of fixed models and random models used in the LMM statistical analyses.

Response variate category	Response variate	Final fixed model	Random model
**Vitamin D status**	25(OH)D_2 concentration	Ewe breed/genotype + ewe age + ewe pre-mating weight	Batch
	25(OH)D_3 concentration	Ewe breed/genotype + ewe pre-mating weight + number of lambs weaned in the last breeding cycle + weaned litter weight in the last breeding cycle	Batch
	25(OH)D concentration	Ewe breed/genotype + ewe pre-mating weight + number of lambs weaned in the last breeding cycle + weaned litter weight in the last breeding cycle	Batch
**Ewe breeding outcomes**	Number of lambs born	25(OH)D_2/25(OH)D_3/25(OH)D concentration + ewe genotype + ewe age + management system + pre-study system + ewe pre-mating weight + number of lambs weaned in the last breeding cycle + first winter feeding	Batch + ram group
	Number of lambs weaned	25(OH)D_2 concentration + ewe genotype + ewe age + management system + pre-study system + ewe pre-mating weight + ewe pre-mating CS + number of lambs weaned in the last breeding cycle + first winter feeding	Batch + ram group
		25(OH)D_3/25(OH)D concentration + ewe genotype + management system + pre-study system + ewe pre-mating weight + ewe pre-mating CS + number of lambs weaned in the last breeding cycle + first winter feeding	Batch + ram group
**Ewe litter weights to weaning (dam dependent)**	Singleton birth weight	25(OH)D_2 concentration + ewe genotype + management system + first winter feeding + ewe pre-mating weight	Batch + ram group
		25(OH)D_3/25(OH)D concentration + ewe genotype + management system + first winter feeding	Batch + ram group
	Singleton marking weight	25(OH)D_2/25(OH)D_3/25(OH)D concentration + ewe genotype + management system + first winter feeding + ewe pre-mating weight	Batch + ram group
	Singleton weaning weight	25(OH)D_2/25(OH)D_3/25(OH)D concentration + ewe genotype + first winter feeding + ewe pre-mating weight	Batch + ram group
	Twin litter birth weight	25(OH)D_2 concentration + ewe genotype + ewe age + pre-study system + ewe pre-mating weight + ewe pre-mating CS	Batch + ram group
		25(OH)D_3/25(OH)D concentration + ewe genotype + management system + ewe pre-mating weight + ewe pre-mating CS	Batch + ram group
	Twin litter marking weight	25(OH)D_2/25(OH)D_3/25(OH)D concentration + ewe genotype + management system + first winter feeding + ewe pre-mating weight + ewe pre-mating CS	Batch + ram group
	Twin litter weaning weight	25(OH)D_2/25(OH)D_3/25(OH)D concentration + ewe genotype + management system + first winter feeding + ewe pre-mating weight + ewe pre-mating CS	Batch + ram group
